# Complete Genome Sequences of Two Porcine Circovirus Type 2 Field Isolates Bearing an Unusual Sequence Duplication in the *rep* Gene

**DOI:** 10.1128/genomeA.00447-14

**Published:** 2014-07-10

**Authors:** Hermann Willems, Regina Hofmeister, Gerald Reiner

**Affiliations:** Department of Clinical Sciences, Justus-Liebig-University Giessen, Giessen, Germany

## Abstract

Porcine circovirus type 2 (PCV-2) is the etiologic agent of porcine circovirus-associated disease (PCVAD). PCV-2 is classified into three genotypes. Here, we present the complete genomic sequences of two PCV-2 isolates (KM and H026) with an unusual sequence duplication in the *rep* gene coding for viral replicase proteins.

## GENOME ANNOUNCEMENT

Porcine circovirus type 2 (PCV-2) is involved in a great number of diseases in swine, such as postweaning multisystemic wasting syndrome (PMWS) (1), porcine dermatitis, and nephropathy syndrome (PDNS) (2), as well as respiratory (3) and reproductive (4) disorders, which are summarized as porcine circovirus-associated diseases (5). PCV-2 is found worldwide and causes considerable economic losses in the pig industry.

PCV-2, a member of the family *Circoviridae*, consists of a single-stranded circular chromosome of 1,766 to 1,768 nucleotides. Up to 11 open reading frames (ORFs) are described (6) for PCV-2, but only three of them are functionally characterized. ORF1 codes for viral replication proteins (Reps), ORF2 for the viral capsid (Cap), and ORF3, which is embedded within ORF1, for an apoptotic protein. PCV-2 isolates are classified into three different genotypes (7), PCV-2a (accession no. AF055392), PCV-2b (accession no. AF055394), and PCV-2c (accession no. EU148503).

PCV-2 isolates KM and H026 were obtained from the lungs of vaccinated pigs originating from two different farms. The genomes of KM and H026 were partially amplified by using two different PCRs. Amplicons with sizes of 824 and 1,173 bp for KM and H026, respectively, were concatenated through their overlapping sequences. KM and H026 belong to the PCV-2b genotype and have a size of 1,767 bp and G+C contents of 48.73 and 49.01%, respectively. The sequence identity to the PCV-2b reference strain is 97.6%. The ORF2 and ORF3 nucleotide and amino acid (aa) sequences are highly similar to those of PCV-2b, with similarities ranging from 98.2 to 99.3%. However, due to a sequence duplication, ORF1 shares only 96.5% of the nucleotides and 94.5 to 95.2% of the amino acids with those of PCV-2b. Ten nucleotides directly upstream ORF1, containing the hexanucleotide motif H4 (CAGCAG) of the replication origin, and the first 34 nucleotides of ORF1 are repeated at position 747 of ORF1. Sequence duplication results in a unique amino acid composition of the Rep protein at position 251 without modifying protein length.

Strikingly, as determined with the protein modeling package I-Tasser (8–10), this unique amino acid composition does not alter the conformation of the Rep protein. Upon transcriptional analysis of PCV-2, Cheung (11) observed a pattern of five viral Rep-associated RNAs (Rep, Rep′, Rep3a, Rep3b, and Rep3c), with all but Rep generated by alternate splicing. Interestingly, alternate transcription from the sequence duplication at position 757 of ORF1 would generate a truncated Rep protein of 62 aa, which is identical to Rep3a (80 aa) and Rep3b (75 aa), except for 2 aa and deletions of 18 and 13 aa, respectively. Whereas the sequences coding for Rep3a and Rep3b depend on the splicing apparatus of the host, the sequences of the truncated Rep protein do not. Obviously, the shift in PCV-2 sequences during the past decades applies not only to whole-genome sequences but also to selective sequence regions. Whether this is also true for sequences of the PCV-2 isolates KM and H026 has yet to be evaluated.

### Nucleotide sequence accession numbers.

The genome sequences of PCV-2 KM and H026 have been assigned GenBank accession no. KJ679445 and KJ679446, respectively.
